# Comparative Analysis of Mitogenomic and Nuclear Gene Data Reveals Phylogenetic Implications, Divergence Times, and Historical Biogeography in the Subfamily Pyrginae (Lepidoptera: Hesperiidae)

**DOI:** 10.1002/ece3.71757

**Published:** 2025-07-13

**Authors:** Jintian Xiao, Xiangyu Hao, Hideyuki Chiba, Yiping Li, Xiangqun Yuan

**Affiliations:** ^1^ Key Laboratory of Plant Protection Resources and Pest Management, Ministry of Education, Entomological Museum, College of Plant Protection Northwest A&F University Yangling China; ^2^ B.P. Bishop Museum Honolulu Hawaii USA; ^3^ Key Laboratory of Integrated Pest Management on Crops in Northwestern Loess Plateau, Ministry of Agriculture, College of Plant Protection Northwest A&F University Yangling China

**Keywords:** divergence time, historical biogeography, mitogenomes, nuclear genes, phylogenetic

## Abstract

The subfamily Pyrginae *sensu lato* (Lepidoptera: Hesperiidae) represents a group of skipper butterflies, consisting of 1132 species in 153 genera and nine tribes. Although the phylogeny and morphology of Pyrginae have been extensively studied, there is limited information on their historical biogeography. Evolutionary relationships among hesperiid subfamilies and tribes are prerequisites for inferring their biogeographic patterns. In this study, the complete mitochondrial genomes of 10 Hesperiidae, in which seven Pyrginae, were newly sequenced. Our sampling encompassed 118 species, including 93 Pyrginae, representing nine tribes and 85 genera. A concatenated super‐matrix of mitogenomic data and the nuclear genes *EF‐1α* and *Wingless* was constructed. The monophyly of Pyrginae was robustly supported (PP = 1, BP = 89). Within Pyrginae, relationships among Tagiadini, Pyrrhopygini, Jerini, and Celaenorrhinini were stable, while relationships among Pyrgini, Carcharodini, Erynnini, and Achlyodidini varied, requiring further study. The most recent common ancestor of Pyrginae *sensu lato* was inferred to have been distributed in the Neotropical region or the Neotropical region + the Palearctic region in the early Paleocene (ca. 56.31 Ma) while that of Pyrginae *sensu stricto* (Achlyodidini, Pyrgini, Carcharodini, and Erynnini) was in the Neotropical region in the early Eocene (ca. 46.72 Ma).

## Graphical abstract

This study examined the phylogeny and historical biogeography of the skipper butterfly subfamily Pyrginae using mitochondrial and nuclear gene data from 118 species. The monophyly of Pyrginae was strongly supported, though some tribal relationships within the subfamily remain unresolved. Biogeographic analysis suggests the most recent common ancestor of Pyrginae *sensu lato* originated in the Neotropical region or Neotropical + Palearctic regions in the early Paleocene, while Pyrginae *sensu stricto* originated in the Neotropics in the early Eocene.

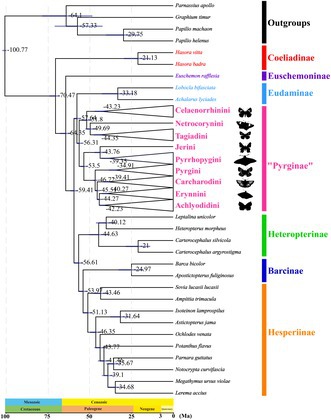

## 1 Introduction

The family Hesperiidae, or skippers, are a unique group of butterflies, with more than 4200 described species (van Nieukerken et al. 2011). Unlike other butterflies, their antennal bases are distantly separated and the antennal tips in most, if not all, species are hooked and named apiculus, significantly different from other butterflies. Evans (1937, 1949) recognized four subfamilies in the old world: Coeliadinae, Pyrginae, Trapezitinae, and Hesperiinae, based on larval food plants, position of the wings at rest, and morphological characters such as labial palpus and wing venation. He then recognized four subfamilies in the new world: Pyrrhopyginae, Pyrginae, Hesperiinae, and Megathyminae, based on male genitalia, antennal shapes, and wing venation (Evans 1951, 1952, 1953, 1955). Recent studies on the phylogeny and taxonomy of Hesperiidae resulted in significant changes in the subfamily classification. Warren et al. (2009), in their pioneering work on skipper phylogeny, combined molecular data (*COI*, *COII*, *EF‐1α*, and *Wingless*) with morphological and biological characters of 209 species in 198 genera to propose a taxonomic framework that recognized seven subfamilies: Coeliadinae, Euschemoninae, Eudaminae, Pyrginae, Heteropterinae, Trapezitinae, and Hesperiinae. Euschemoninae and Eudaminae were considered distinct from Pyrginae while Pyrrhopyginae *sensu* Evans (1951) was included in Pyrginae *sensu* Evans (1949, 1953), and Megathyminae was merged into Hesperiinae. In their phylogeny, Pyrginae is sister to Heteropterinae + Trapezitinae + Hesperiinae, and Eudaminae is branched out before that cluster. Sahoo et al. (2016), using 311 species in 270 genera, found two alternative relationships of Eudaminae and Pyrginae: one is identical to Warren et al. (2009) and, in the other phylogeny, Coeliadinae, Euschemoninae, and Eudaminae are trifurcated. In both Warren et al. (2009) and Sahoo et al. (2016), the traditionally defined Pyrginae is polyphyletic. Li et al. (2019) used the low‐coverage whole genome sequencing data of 250 species in 186 genera to propose a taxonomic framework with nine subfamilies: Coeliadinae, Euschemoninae, Eudaminae, Tagiadinae, Pyrrhopyginae, Pyrginae, Heteropterinae, Trapezitinae, and Hesperiinae. In addition to recognizing Euschemoninae and Eudaminae as distinct, Tagiadinae (Tagiadini and Celaenorrhini) was treated as a distinct subfamily and Pyrrhopygini *sensu* Warren et al. (2009) was reinstated as a distinct subfamily Pyrrhopyginae. In their phylogeny, as well as that of Toussaint et al. (2018), Pyrginae and Eudaminae are sister groups forming a monophyletic cluster. Phylogenetic relationships can test the monophyly of a group but do not automatically assign that group to any particular taxonomic category. Therefore, it can be said that the assignment is merely a taxonomic decision to decide whether the group is a subfamily, tribe, subtribe, or other taxonomic categories. Li et al. (2019), Cong et al. (2019), Zhang et al. (2019a, 2019b), and subsequent studies of the same research group used the depth of branch as a criterion as to which taxonomic category a monophyletic group belongs without much attention to morphology. In the present study, we follow the concept of Pyrginae used by Warren et al. (2009) in order to retain a lesser number of subfamilies.

The subfamily Pyrginae *sensu* Warren et al. (2009) (Pyrginae *sensu lato* hereafter) consists of 1132 species in 153 genera (Ferrer‐Paris et al. 2013) and is distributed nearly worldwide. The members share the following morphological characters: the vein M2 of the forewing is closer to the vein M1 and far from the vein M3; the third segment of the labial palpus is usually short and thick; the abdomen is shorter than or equal to the posterior margin of the hindwing, not extending beyond the anal angle of the hindwing; there is no obvious tail on the hindwing; the larvae of Pyrginae mainly feed on dicots, such as Verbenaceae and Poaceae (Sahoo et al. 2017).

In Pyrginae *sensu lato*, we recognize nine tribes: Celaenorrhini, Netrocorynini, Tagiadini, Pyrrhopygini, Jerini, Achlyodidini, Carcharodini, Pyrgini, and Erynnini. Pyrginae *sensu* Li et al. (2019) (Pyrginae *sensu stricto* hereafter) includes four tribes: Achlyodidini, Carcharodini, Pyrgini, and Erynnini.

There has been inconsistent results regarding the divergence time of the subfamily Pyrginae. Espeland et al. (2018) constructed a phylogenetic framework for Lepidoptera based on fossil calibrations and showed that the crown group of *Pyrginae sensu lato* diverged in the early Paleocene (ca. 55 Ma). However, only seven species in seven genera of Pyrginae *sensu lato* were used in this analysis. Chazot et al. (2019) estimated, in the study including 76 pyrgine species in 75 genera, the divergence times of butterflies (Papilionoidea) and showed that the earliest radiation of crown group of Pyrginae was the mid‐Paleocene (ca. 45 Ma). Li et al. (2019), using low coverage whole‐genome sequencing and including 86 species in 86 genera, estimated the crown group of Pyrginae to have diverged in the early Paleocene (ca. 55 Ma) as in Espeland et al. (2018). Kawahara et al. (2023), using 391 genes of 370 species in 145 genera captured by anchored hybrid enrichment method, also estimated the crown group of Pyrginae to have diverged in the early Paleocene but a little earlier (ca. 55.5 Ma), based on data from 370 species in 145 genera. Differences in divergence time estimates may be due to differences in taxon sampling, choice of molecular markers, and analytical methods.

Given the global distribution of the subfamily Pyrginae, exploring their biogeography and evolution is needed to understand their complex speciation processes and historical biogeography. Considering the lack of comprehensive and systematic research on pyrgine biogeography, robust estimates for their divergence time, ancestral area reconstructions, and phylogeny are all required.

In this study, we reconstruct a molecular phylogeny of 85 genera (approximately 56% of the total 153 genera) in Pyrginae based on mitogenomes and two nuclear genes (*EF‐1α* and *Wingless*) to address these questions.

## 2 Materials and Methods

### 2.1 Sampling and DNA Extraction

Our samples included 114 hesperiid species, among which 93 were Pyrginae representing nine tribes and 85 genera. Four species of Papilionidae (*Graphium timur*, *Papilio machaon* , *P. helenus*, and *Parnassius apollo*) were selected as outgroups.

Ten mitochondrial genomes were newly sequenced, including seven species in six genera from the subfamily Pyrginae. Collection localities for samples are listed in Table 1. Field collected adult specimens were preserved in anhydrous ethanol. Ethanol was replaced within a week of collection, and the samples were stored in a cool environment. After returning to the laboratory, ethanol was replaced again, and specimens were stored at −20°C. Taxonomic identification was conducted using morphological characteristics, particularly genitalia, and subsequently confirmed using *cox1* barcode obtained from the BOLD database (Macher et al. 2017). DNA was extracted from thoracic muscle tissue using the Biospin Insect Genomic DNA Extraction Kit (Qiagen, Hilden, Germany). An additional 81 mitochondrial genomes were assembled and annotated from the SRA data.

**TABLE 1:** Collection information of adult specimens in this study.

Species	Collection spot	Collection data	Tribes	Accession numbers
*Celaenorrhinus consanguineus*	Wutaishan, Shanxi, China	2020.8	Celaenorrhini	OR024665
*Celaenorrhinus maculosus*	Mount Emei, Sichuan, China	2020.8	Celaenorrhini	OR024663
*Coladenia agnioides*	Nanling Mountains, Guangdong, China	2020.8	Tagiadini	OR045388
*Pintara bowringi*	Dupang Mountain, Hunan, China	2020.7	Tagiadini	OR004471
*Pyrgus alveus speyeri*	Yuzhong, Gansu, China	2021.7	Pyrgini	OR004470
*Erynnis pelias*	Yuzhong, Gansu, China	2020.8	Erynnini	OR004469
*Sloperia tessellum dilutior*	Songjianghe, Jilin, China	2021.7	Pyrgini	OR045387
*Ampittia trimacula*	Yuzhong, Gansu, China	2017.8	Aeromachini	OR248671
*Sovia lucasii lucasii*	Baixiongping, Sichuan, China	2021.8	Aeromachini	OR024664
*Carterocephalus argyrostigma*	Yuzhong, Gansu, China	2017.5	Carterocephalini	OR045386

### 2.2 Bioinformatics Analysis

The 10 newly collected samples were sequenced using the Illumina HiSeq 2000 system at Genesky Biotechnologies Inc. (Shanghai, China) for 150 bp paired‐end reads. All samples generated approximately 8 GB of paired‐end data, except *Sloperia* and *Pintara*, which yielded approximately 3 GB of data per sample. Initial analysis and quality control were performed using CLC Genomic Workbench v10.0 (CLC Bio, Aarhus, Denmark) with default settings. Clean reads were assembled and annotated using MitoZ v3.6 with the “all” parameter for automated processing and the “‐‐requiring_taxa” Arthropodaoption to specify the target taxonomic group (Meng et al. 2019). Sequence alignment was performed using the MAFFT plugin as integrated in Geneious 9.0.2 (Kearse et al. 2012; Katoh and Standley 2013). The identification of RNAs was performed using the MITOS Web Server (accessed on February 1, 2022) (Bernt et al. 2013).

### 2.3 Sequence Read Archive (SRA) Data Extraction

Eighty one SRA files containing low‐coverage whole genome data for additional hesperiids were downloaded from GenBank to assemble mitogenomes. Subsequent annotation and assembly of the raw data were performed using Geneious 9.0.2 (Kearse et al. 2012). SRA data were also used to assemble *EF‐1α* and *Wingless* genes using Geneious 9.0.2. Details of the source of the SRA data and the accession number of the mitochondrial genome data obtained from GenBank are provided in Table S1. Details of the source of the *EF‐1α* and *Wingless* genes are provided in Table S2.

### 2.4 Phylogenetic Reconstruction

Both partitioned maximum likelihood (ML) and Bayesian inference (BI) methods were applied to the PRTN dataset. We assembled one dataset for phylogenetic analysis: a PRTN matrix containing 13 PCGs, RNA genes, and nuclear genes (14,710 bp). We used MrBayes 3.2.7a (Ronquist et al. 2012) and IQ‐TREE v1.6.0 (Kalyaanamoorthy et al. 2017) for phylogenetic reconstruction. We inferred the species tree using ASTRAL‐III (Zhang et al. 2018). Specifically, we constructed individual gene trees for each of the 13 protein‐coding genes and 2 nuclear genes using IQ‐TREE. Additionally, we combined the sequences of 22 tRNA and 2 rRNA genes into a single dataset to construct one gene tree. In total, this approach yielded 16 gene trees, which were then utilized by ASTRAL‐III to infer the species tree.

Model selection for each partition was performed using ModelFinder, implemented in IQ‐TREE v1.6.0. The best partitioning schemes and models for Maximum likelihood (ML) method and Bayesian inference (BI) methods based on PRTN dataset are shown in Tables S4 and S5. Whole mitogenomes were extracted using PhyloSuite v1.2.2 (Zhang et al. 2020). Sequences of 13 PCGs from 123 species were incorporated into PhyloSuite for batch alignment through MAFFT (Katoh and Standley 2013). We used the G‐INS‐i strategy and codon alignment mode to align the 13 PCGs sequences. All RNAs were aligned using MAFFT with the Q‐INS‐i strategy. Gblocks v0.91b (Castresana 2000) was used through PhyloSuite v1.2.2 to eliminate ambiguous sites with default parameters. Individual genes were concatenated using PhyloSuite v1.2.2.

### 2.5 Divergence Time Estimate

Given the lack of any pyrgine fossil record, we used the MCMCtree module in the PAML v4.9j software to estimate divergence time by setting secondary calibrations (Yang 2007). The root node of this tree was estimated to be 101 million years, as proposed by Kawahara et al. (2023) for the divergence time of the Papilionoidea. Three additional secondary calibrations were also used from Kawahara et al. (2023): (1) the crown age of Hesperiidae was set at 73.34 Ma with a highest posterior density (HPD) range of 68.21–75.18 Ma; (2) the crown age of Pyrginae *sensu lato* was set at 55.50 with a (HPD) range of 50.28–57.22 Ma; (3) the crown age of the clade (Carcharodini + Pyrgini + Erynnini + Achlyodidini) was set at 44.22 Ma with a (HPD) range of 39.87–46.99 Ma. For dating analysis, the IQ tree derived from the PRTN datasets was used as the input tree in MCMCtree in PAML v4.9j (Yang 2007) using likelihood approximation along with gradient and Hessian matrix computation of the branch lengths to hasten computation. We analyzed the dataset under the REV substitution model with gamma, incorporating five rate parameters. The clock was configured as independent, the gamma prior for the transition/transversion ratio (kappa) was set to *a* = 6, *b* = 2, the gamma prior for variable rates among sites (alpha) to *a* = 1, *b* = 1, the tree prior was designated as birth‐death, and the rate prior was lognormal. To ensure the validity of the priors, we initially performed an analysis without likelihood computation by setting use data = 0. Sequence types were set as nucleotides and independent rates were selected for the clock model. A total of 50,000 trees were burned‐in with a sampling frequency of once every 50 trees, eventually retaining 20,000 trees. Median ages and 95% highest posterior density (HPD) intervals for node‐ages were visualized with FigTree v1.4.5 (http://tree.bio.ed.ac.uk/software/figtree/).

### 2.6 Historical Biogeography

We estimated the ancestral range of the subfamily Pyrginae using the Reconstructing Ancestral State in Phylogenies (RASP: Yu et al. 2020) based on the divergence time trees generated by MCMCtree implemented in the PAML v4.9j software (Yang 2007). Ancestral range was estimated by the S‐DEC model that allows for sympatric speciation, allopatric speciation, and anagenetic range expansion and contraction (Ree et al. 2005; Beaulieu et al. 2013). We aggregated global distribution data at the species level from two resources: (1) the Global Biodiversity Information Facility (GBIF) (https://www.gbif.org/) and (2) WikiSpecies (https://species.wikimedia.org). We set the maximum number of ancestral areas to two and all other parameters to default. We coded Pyrginae distribution into six areas: (A) Neotropical region, (B) Nearctic region, (C) Palearctic region, (D) Afrotropical region, (E) Indo‐Malayan region, and (F) Australasian region. The geographic information for each species is provided in Table S3.

## 3 Results

### 3.1 Phylogenetic Relationships

**FIGURE 1:**
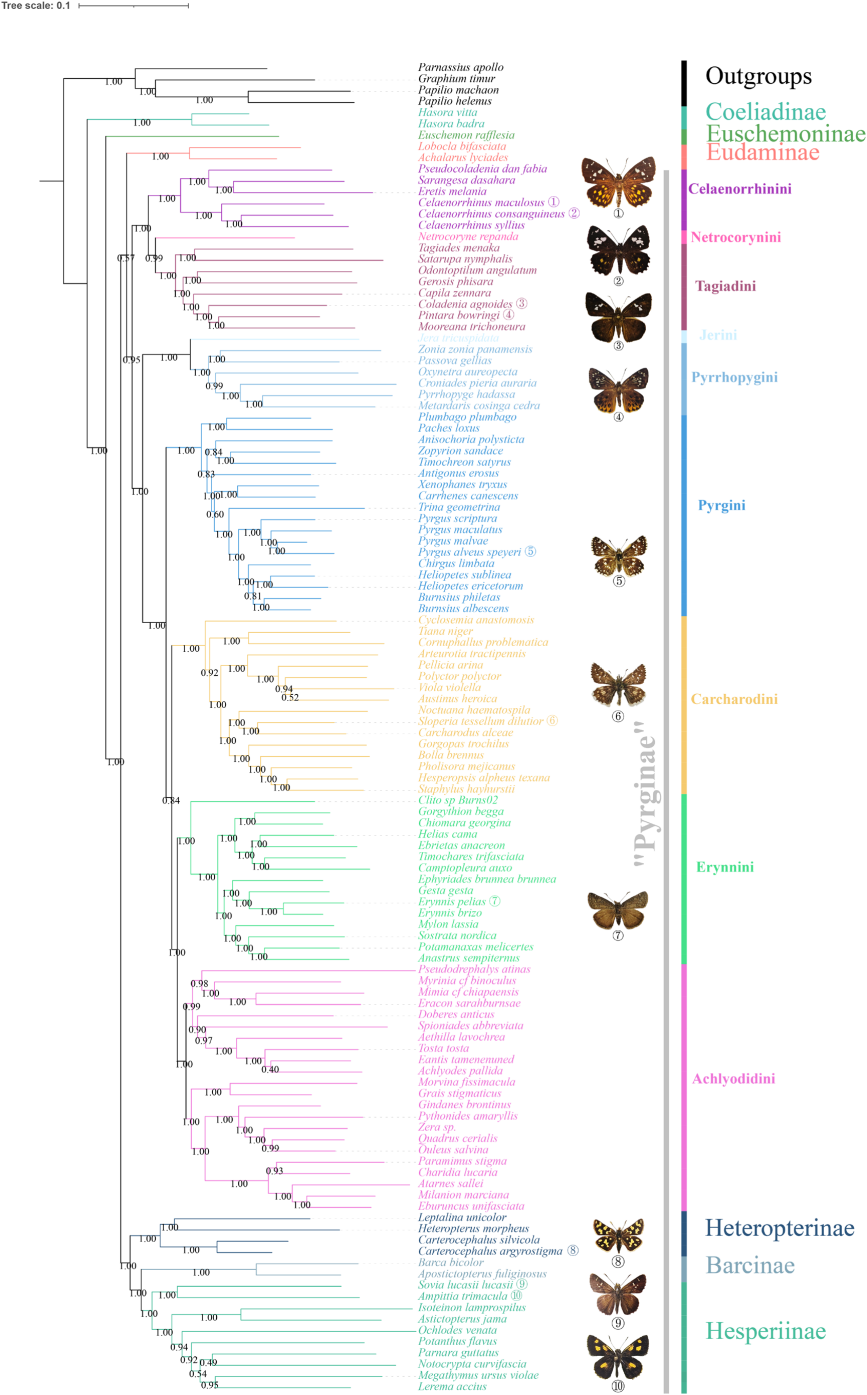
Phylogenetic trees inferred from the PRTN dataset using MrBayes.

**FIGURE 2:**
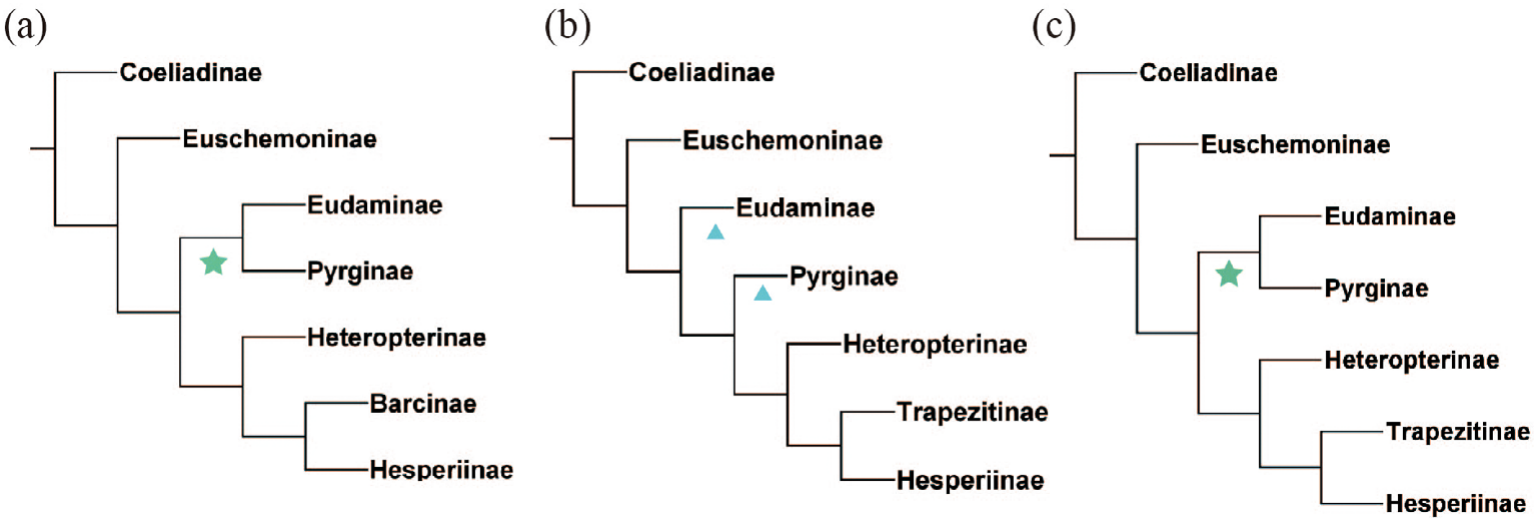
Two hypotheses of the phylogenetic relationship of subfamilies in the family Hesperiidae. (a) This study, (b) Warren et al. (2009) and Sahoo et al. (2016), (c) Toussaint et al. (2018) and Li et al. (2019). 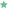 Indicates a monophyletic clade. 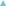 Indicates two clades that are paraphyletic in relation to each other.

Phylogenetic trees obtained by the ML and BI inference were congruent. The phylogenetic tree based on Bayes analysis of the PRTN data set is shown (Figure 1), and the remaining analyses are found in Figures S1 and S2. The monophyly of the subfamily Pyrginae was highly supported in both methods (PP = 0.972, BP = 100), with Eudaminae as the sister group of Pyrginae. Phylogenetic relationships at the subfamily level were consistent with Toussaint et al. (2018), Li et al. (2019), and Kawahara et al. (2023), but differed from those of Warren et al. (2009) and Sahoo et al. (2016). This may be due to different dataset compositions. Although we used far less number of genes, the result was identical to those research studies using extensive amounts of datasets, suggesting that the size of the dataset, at least at the subfamily level, does not really matter. These alternative phylogenetic relationships are shown in Figure 2. The subsequent analysis was carried out using the BI tree. In this study, we inferred the species tree, but its topology did not show congruence with the previous studies, particularly in the relationships among subfamilies. Therefore, the species tree was not used for further analyses in this study. In the topological structure testing performed by the AU test, the ML tree achieved a higher *p*‐value. The *p*‐AU value for the ML tree is 0.587, while that for the BI tree is 0.413, with both trees receiving strong support in the significance testing of their topologies. Nevertheless, the BI tree shows better support values at certain tribal‐level nodes. Given the absence of substantial topological differences between the two trees, subsequent phylogenetic analyses were conducted using the BI tree. The results of the AU test are shown in Table S6.

Seven monophyletic tribes were recognized within Pyrginae *sensu lato*, with two monotypic tribes. Celaenorrhinini was the sister group to Netrocorynini + Tagiadini (PP = 1). Pyrrhopygini + Jerini was the sister group of Pyrginae *sensu stricto* (PP = 1). The relationships among four tribes within Pyrginae *sensu stricto* were as follows: Pyrgini + (Carcharodini + (Erynnini + Achlyodidini)). Relationships among Celaenorrhinini, Netrocorynini, Tagiadini, Pyrrhopygini, and Jerini were consistent with Li et al. (2019).

### 3.2 Divergence Times and Ancestral Range

**FIGURE 3:**
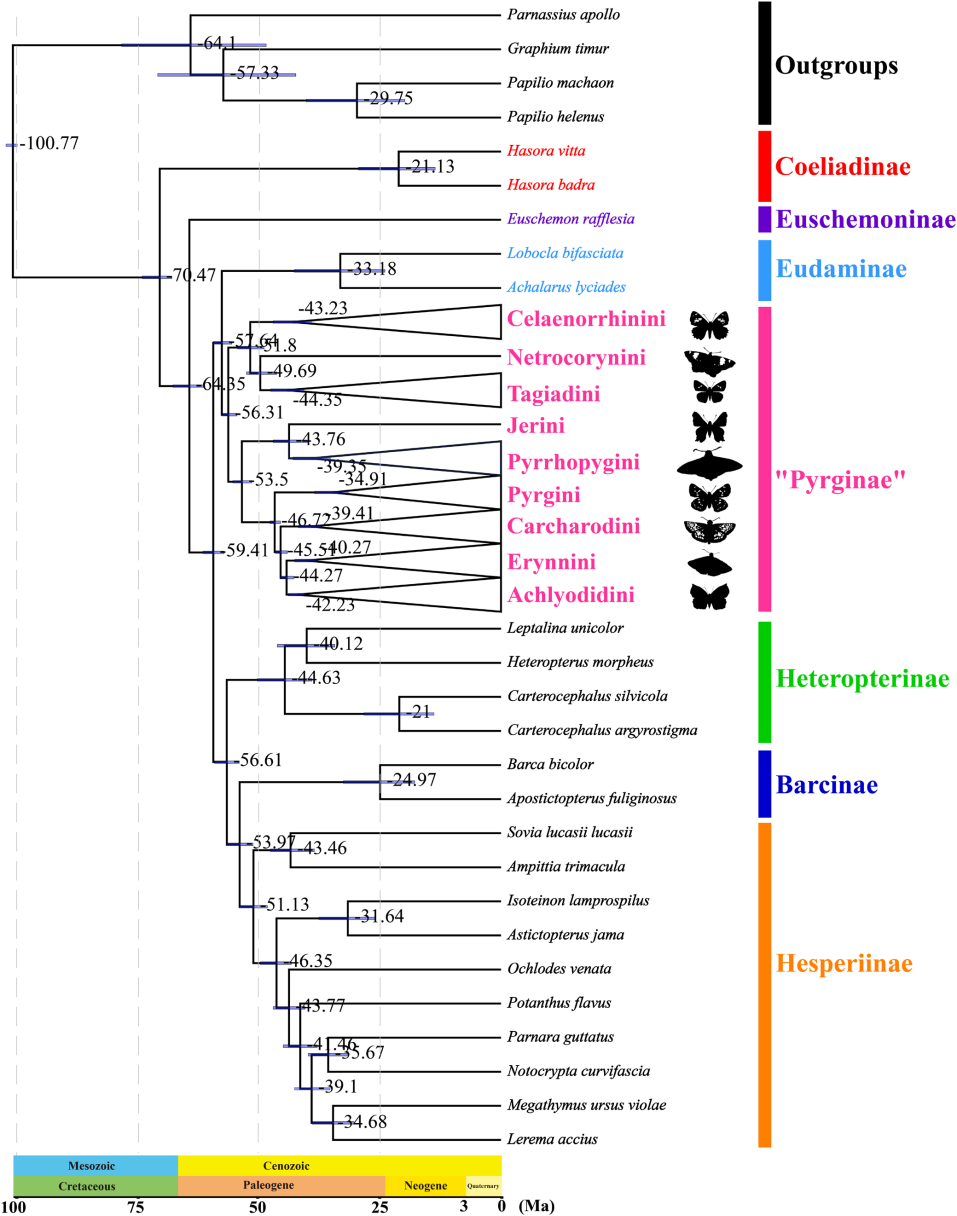
The divergence times of the phylogenetic tree based on PRTN dataset using Bayesian analysis.

Estimated divergence times are shown in Figure 3. The initial divergence of Pyrginae *sensu lato* occurred in the late Paleocene, ca. 56.31 Ma (53.53–57.61 Ma, 95% HPD). For the tribes within Pyrginae *sensu lato*, the divergence of all the tribes mainly took place during the Eocene at 43.23 Ma (39.19–47.03 Ma, 95% HPD) for Celaenorrhinini; at 44.35 Ma (40.69–47.55 Ma, 95% HPD) for Tagiadini; at 39.35 Ma (35.24–42.96 Ma, 95% HPD) for Pyrrhopygini; at 39.41 Ma (37.05–41.81 Ma, 95% HPD) for Carcharodini; at 34.91 Ma (31.58–38.45 Ma, 95% HPD) for Pyrgini; at 40.27 Ma (37.87–42.59 Ma, 95% HPD) for Erynnini; at 42.23 Ma (40.54–43.88 Ma, 95% HPD) for Achlyodidini. The 95% HPD ranges for node ages and detailed node ages are shown in Figures S3 and S4.

**FIGURE 4:**
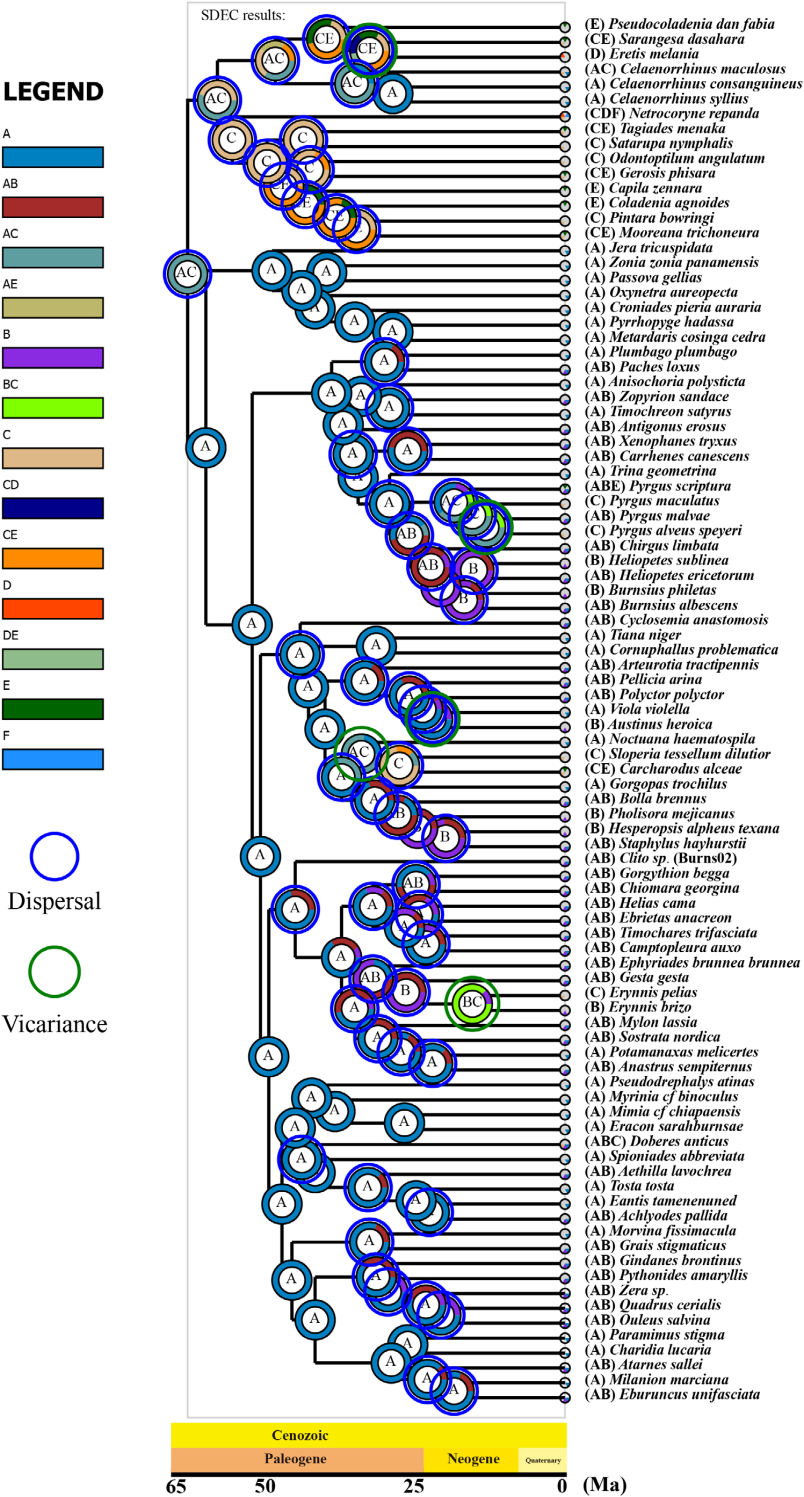
Biogeographic history of Pyrginae *sensu lato* using RASP v4.2 in S‐DEC model. The letter at the node indicates the distribution of most recent common ancestor. The blue circle of the node indicates the dispersal events, green circle indicates the vicariance events, and yellow circle indicates the extinction events.

Ancestral range inferences are shown in Figure 4, which infers 57 dispersal events and 5 vicariance events. The results indicate that the most recent common ancestor of the subfamily Pyrginae *sensu lato* was distributed in the Neotropical region or the Palearctic region, while the most recent common ancestor of subfamily Pyrginae *sensu stricto* was distributed in the Neotropical (Figure 4).

## 4 Discussion

### 4.1 Systematics of Pyrginae

**FIGURE 5:**
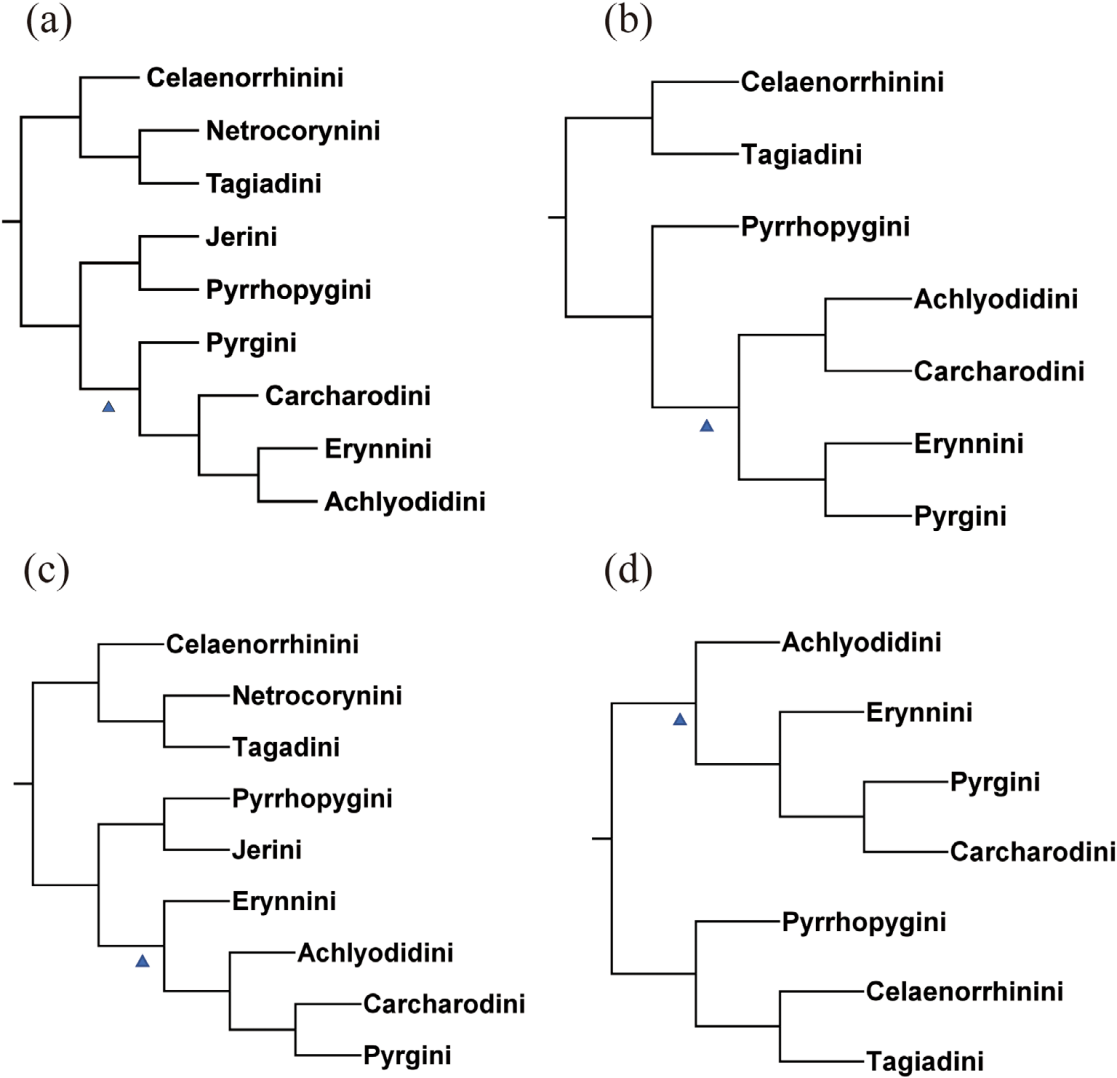
Different phylogenetic hypotheses within Pyrginae *sensu lato*. (a) This study, (b) Toussaint et al. (2018), (c) Li et al. (2019) and (d) Huang et al. (2024). 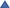 indicates the phylogenetic relationships of the four tribes within Pyrginae *sensu stricto*.

In this study, a comprehensive species sampling of Pyrginae *sensu lato* was conducted. The resulting relationships among tribes within Pyrginae *sensu stricto* are not consistent with previous studies. Our result shows that the topology of these four tribes is delineated as follows: Pyrgini + (Carcharodini + (Erynnini + Achlyodidini)). Whereas in Toussaint et al. (2018), the topology of these four tribes is delineated as follows: (Achlyodidini + Carcharodini) + (Erynnini + Pyrgini), while in Li et al. (2019), the topology is: Erynnini + (Achlyodidini + (Carcharodini + Pyrgini)), and in Huang et al. (2024), Achlyodidini + (Erynnini + (Pyrgini + Carcharodini)). Different phylogenetic hypotheses within Pyrginae *sensu lato* as reported in Toussaint et al. (2018), Li et al. (2019), and Huang et al. (2024), are shown in Figure 5. The relationships of these four tribes, despite studies using molecular markers of multiple gene segments, have not yielded consistent results with those constructed using genome‐wide data or anchored hybridization enrichment (AHE) data. These inconsistencies are likely due to the use of different analytical techniques, taxon sampling, and gene choice. Recent studies have highlighted the importance of integrating multiple types of molecular data to resolve complex phylogenetic relationships in various taxonomic groups (McCormack et al. 2013; Anderson et al. 2023). The combination of traditional Sanger sequencing data with high‐throughput sequencing approaches, such as target enrichment and whole‐genome sequencing, has been shown to improve phylogenetic resolution and support across different evolutionary timescales (Faircloth et al. 2012; Bravo et al. 2019). This integrative approach may be particularly valuable for resolving challenging phylogenetic relationships within Pyrginae and other lepidopteran groups.

Our research added further knowledge by integrating two data sources, mitochondrial genomes and nuclear genes. The relationships among four tribes within Pyrginae *sensu stricto* need further study, and integrative analysis combining molecular and morphological data should be conducted to construct the phylogenetic tree.

### 4.2 Historical Biogeography

The Neotropical region became a diversification center for many biological groups (Gómez‐Díaz et al. 2023). Biodiversity changes following the end‐Cretaceous mass extinction may have provided an opportunity for the early origin and evolution of certain insect groups as discussed by Pires et al. (2018). After the K‐Pg event, the Earth experienced a warm period with higher global temperatures, especially the absence of permanent ice sheets at high latitudes (Cramwinckel et al. 2018) created favorable conditions for the expansion of many tropical and subtropical species (Zachos et al. 2008). This warm global climate facilitated the dispersal and diversification of many species, probably including butterflies (Kawahara et al. 2023).

Our results suggest that the divergence of the Celaenorrhinini took place during the mid‐Eocene, at ca. 43.23 Ma (Figure 3). Ancestral range reconstruction analyses suggested that the most recent common ancestor of Celaenorrhinini was likely in the Neotropical region or Palearctic region. The period from ca. 56 to 33.9 Ma encompassed major changes in Earth's biodiversity and climate (Su et al. 2019; Kad et al. 2022). During this period, global temperatures were 9°C–14°C higher than present (Kad et al. 2022). These changes appear to have led to a rapid diversification of Pyrginae tribes. Within the Tagiadini, the most recent common ancestor of the clade *Tagiades* + *Satarupa* originated in the Palearctic region. While the ancestor of the major Tagiadini clade (*Odontoptilum*, *Gerosis*, *Capila*, *Coladenia*, *Pintara*, and *Mooreana*) also originated in the Palearctic region or Indo‐Malayan region from ca. 42.05 Ma (Figure S4). The common ancestor of the tribes Pyrrhopygini and Jerini originated in the Neotropical region, and all the genera are still found in the Neotropical (Figure 4). During the Eocene, the warm global climate and smaller temperature gradients promoted the rapid diversification of species, especially mammals and flowering plants (Gingerich 2006; Jaramillo et al. 2006), and the latter have been proposed to affect the diversity of butterfly species (Gordon and Kerr 2022).

The most recent common ancestor divergence time of four tribes within Pyrginae *sensu stricto* is as follows: Pyrgini (ca. 34.91 Ma), Achlyodidini (ca. 42.23 Ma), Carcharodini (ca. 39.41 Ma), and Erynnini (ca. 40.27 Ma) (Figure 3). Due to higher temperatures in these periods than in the late Paleocene, most of the genera of these four tribes colonized the Neotropical region, and several genera that diverged later dispersed to the Nearctic region and Palearctic region (Zachos et al. 2008).

### 4.3 Concluding Remarks

Our study provides a detailed analysis of the subfamily Pyrginae, integrating phylogenetic, divergence time, and historical biogeography to reveal patterns of diversification and migration within the group, providing new insights into its evolutionary dynamics and biogeographic distribution. Although we merely used mitochondrial genomic data and two nuclear genes (*EF‐1α*, *Wingless*), it confirmed the monophyly of Pyrginae + Eudaminae and is consistent with the results of Li et al. (2019). With all those changes in the traditional concept of Pyrginae using molecular data, it seems that we returned to the morphology‐based group of Pyrginae, including Pyrrhopygini and excluding Euschemonidae, after all. Whether Eudaminae should be treated as a tribe of Pyrginae or retained as is still needs further investigation.

Pyrginae *sensu lato* consists of nine tribes, in which the relationships of Pyrgini, Carcharodini, Erynnini, and Achlyodidini remain to be further investigated, while the relationships of the rest of the tribes are stable. Our results suggest that the most recent common ancestor of Pyrginae *sensu lato* diverged at ca. 56.31 Ma, during the Paleocene epoch in the Neotropical region or the Palearctic region, and that the most recent common ancestor of Pyrginae sensu stricto diverged at ca. 46.72 Ma in the early Eocene in the Neotropical region. This study modestly contributes to the understanding of the evolutionary history and classification of the subfamily Pyrginae, representing a step forward in entomological research.

## Author Contributions

**Jintian Xiao:** conceptualization (equal), data curation (equal), formal analysis (equal), validation (equal), writing – original draft (equal). **Xiangyu Hao:** formal analysis (equal), methodology (equal). **Hideyuki Chiba:** conceptualization (equal), writing – review and editing (equal). **Yiping Li:** formal analysis (equal), supervision (equal), validation (equal). **Xiangqun Yuan:** funding acquisition (equal), investigation (equal), writing – review and editing (equal).

## Conflicts of Interest

The authors declare no conflicts of interest.

## Supporting information


Data S1.

## Data Availability

The following information was supplied regarding the availability of DNA sequences: The complete mitogenomes of *Ampittia trimacula*, *Carterocephalus argyrostigma*, *Celaenorrhinus consanguineus*, *Celaenorrhinus maculosus*, *Coladenia agnioides*, *Erynnis pelias*, *Pintara bowringi*, *Pyrgus alveus speyeri*, *Sloperia tessellum dilutior*, and *Sovia lucasii lucasii* are deposited in GenBank of NCBI under accession numbers OR248671, OR045386, OR024665, OR024663, OR045388, OR004469, OR004471, OR004470, OR045387, and OR024664, respectively. All sequences used in this study were submitted to NCBI GenBank, have been released, and received accession numbers. Raw data of 10 newly sequenced mitochondrial genomes have been uploaded to the NCBI GenBank, and the BioProject accession number is PRJNA1073187. All information, including Tables S1–S6 and Figures S1–S4, can be found in the Supporting Information accompanying this article.
