# Supplementary material for: Comparative Analysis of Mitogenomic and Nuclear Gene Data Reveals Phylogenetic Implications, Divergence Times, and Historical Biogeography in the Subfamily Pyrginae (Lepidoptera: Hesperiidae)
Source: Ecol Evol. 2025 Jul 13;15(7):e71757. doi: 10.1002/ece3.71757 (PMC12256114; doi:10.1002/ece3.71757)

Table S1. Species information and GenBank accession numbers

Subfamily	Species	Accession number	Reference
Coeliadinae	*Hasora vitta*	NC_027170	(Cao et al. 2016)
	Hasora badra	NC_045249	Unpublished
Euschemoninae	*Euschemon rafflesia*	NC_034231	(Zhang et al. 2017b)
Eudaminae	*Lobocla bifasciata*	KJ629166	(Kim et al. 2014)
	Cecropterus lyciades	NC_030602	(Shen et al. 2016)
Heteropterinae	*Carterocephalus argyrostigma*	OR045386	This study
	Heteropterus morpheus	NC_028506	Unpublished
	Leptalina unicolour	MK265705	(Jeong et al. 2019)
	Carterocephalus silvicola	NC_024646	(Kim et al. 2014)
Barcinae	*Barca bicolor*	NC_039947	(Han et al. 2018)
	Apostictopterus fuliginosus	NC_039946	(Han et al. 2018)
Hesperiinae	*Ampittia trimacula*	OR248671	This study
	Sovia lucasii lucasii	OR024664	This study
	Isoteinon lamprospilus	NC_042215	(Ma et al. 2020)
	Lerema accius	NC_029826	(Cong and Grishin 2016)
	Notocrypta curvifascia	NC_042216	(Ma et al. 2020)
	Ochlodes venata	NC_018048	Unpublished
	Parnara guttatus	NC_029136	(Shao et al. 2015)
	Astictopterus jama	NC_042214	(Ma et al. 2020)
	Potanthus flavus	NC_024650	(Kim et al. 2014)
	Megathymus ursus violae	KY_630502	(Zhang et al. 2017a)
Pyrginae	*Celaenorrhinus consanguineus*	OR024665	This study
	Celaenorrhinus maculosus	OR024663	This study
	Celaenorrhinus syllius	SRR7174479	(Li et al. 2019)
	Coladenia agnioides	OR045388	This study
	Capila zennara	SRR7174484	(Li et al. 2019)

Subfamily	Species	Accession number	Reference
	Tagiades menka	MZ221164	(Xiao et al. 2022)
	Satarupa nymphalis	MZ221163	(Xiao et al. 2022)
	Eretis melania	SRR7174485	(Li et al. 2019)
	Netrocoryne repanda	SRR7174483	(Li et al. 2019)
	Odontoptilum angulatum	MW381783	(Li et al. 2019)
	Pintara bowringi	OR004471	This study
	Pseudocoladenia dan fabia	SRR7174480	(Li et al. 2019)
	Sarangesa dasahara	SRR7174486	(Li et al. 2019)
	Jera tricuspidata	SRR7174433	(Li et al. 2019)
	Passova gellias	SRR7174439	(Li et al. 2019)
	Zonia zonia panamensis	SRR7174438	(Li et al. 2019)
	Oxynetra aureopecta	SRR7174437	(Li et al. 2019)
	Croniades pieria auraria	SRR7174434	(Li et al. 2019)
	Metardaris cosinga cedra	SRR7174435	(Li et al. 2019)
	Pyrrhopyge hadassa	SRR7174436	(Li et al. 2019)
	Eantis pallida	SRR7174366	(Li et al. 2019)
	Aethilla lavochrea	SRR7174432	(Li et al. 2019)
	Anastrus sempiternus	SRR7174507	(Li et al. 2019)
	Anisochoria polysticta	SRR7174531	(Li et al. 2019)
	Antigonus erosus	SRR7174496	(Li et al. 2019)
	Arteurotia tractipennis	SRR7174564	(Li et al. 2019)
	Atarnes sallei	SRR7174338	(Li et al. 2019)
	Austinus heroica	SRR7174561	(Li et al. 2019)
	Bolla brennus	SRR7174539	(Li et al. 2019)
	Burnsius albescens	SRR7174499	(Li et al. 2019)
	Burnsius philetas	SRR7174498	(Li et al. 2019)
	Camptopleura auxo	SRR7174462	(Li et al. 2019)

Subfamily	Species	Accession number	Reference
	Carcharodus alceae	SRR7174533	(Li et al. 2019)
	Carrhenes canescens	SRR7174490	(Li et al. 2019)
	Charidia lucaria	SRR7174567	(Li et al. 2019)
	Chiomara georgina	SRR7174467	(Li et al. 2019)
	Chirgus limbate	SRR7174488	(Li et al. 2019)
	Clito sp. (Burns02)	SRR7174503	(Li et al. 2019)
	Cornuphallus problematica	SRR7174563	(Li et al. 2019)
	Cyclosemia anastomosis	SRR7174565	(Li et al. 2019)
	Doberes anticus	SRR7174369	(Li et al. 2019)
	Eantis tamenund	SRR7174365	(Li et al. 2019)
	Ebrietas anacreon	SRR7174464	(Li et al. 2019)
	Eburuncus unifasciata	SRR7174345	(Li et al. 2019)
	Ephyriades brunnea brunnea	SRR7174465	(Li et al. 2019)
	Eracon sarahburnsae	SRR7174373	(Li et al. 2019)
	Erynnis brizo brizo	SRR7174469	(Li et al. 2019)
	Erynnis pelias	OR004469	This study
	Gerosis phisara	MZ221157	(Xiao et al. 2022)
	Gesta gesta	SRR7174466	(Li et al. 2019)
	Gindanes brontinus	SRR7174337	(Li et al. 2019)
	Gorgopas trochilus	SRR7174532	(Li et al. 2019)
	Gorgythion begga	SRR7174468	(Li et al. 2019)
	Grais stigmaticus	SRR7174368	(Li et al. 2019)
	Helias cama	SRR7174506	(Li et al. 2019)
	Heliopetes ericetorum	SRR7174500	(Li et al. 2019)
	Heliopetes sublinea	SRR7174501	(Li et al. 2019)
	Hesperopsis alpheus texana	SRR7174536	(Li et al. 2019)
	Milanion marciana	SRR7174344	(Li et al. 2019)

Subfamily	Species	Accession number	Reference
	Mimia cf. chiapaensis	SRR7174372	(Li et al. 2019)
	Mooreana trichoneura	MZ221161	(Xiao et al. 2022)
	Morvina fissimacula	SRR7174367	(Li et al. 2019)
	Mylon lassia	SRR7174502	(Li et al. 2019)
	Myrinia cf. binoculus	SRR7174371	(Li et al. 2019)
	Noctuana haematospila	SRR7174535	(Li et al. 2019)
	Ouleus salvina	SRR7174340	(Li et al. 2019)
	Paches loxus	SRR7174494	(Li et al. 2019)
	Paramimus stigma	SRR7174568	(Li et al. 2019)
	Pellicia arina	SRR7174570	(Li et al. 2019)
	Pholisora mejicanus	SRR7174538	(Li et al. 2019)
	Plumbago plumbago	SRR7174495	(Li et al. 2019)
	Polyctor polyctor	SRR7174562	(Li et al. 2019)
	Potamanaxas melicertes	SRR7174504	(Li et al. 2019)
	Pseudodrephalys atinas	SRR7174341	(Li et al. 2019)
	Pyrgus alveus speyeri	OR004470	This study
	Pyrgus maculatus	NC_030192	Unpublished
	Pyrgus malvae	SRR7174492	(Li et al. 2019)
	Pyrgus scriptura	SRR7174487	(Li et al. 2019)
	Pythonides amaryllis	SRR7174336	(Li et al. 2019)
	Quadrus cerialis	SRR7174343	(Li et al. 2019)
	Sloperia tessellum dilutior	OR045387	This study
	Sostrata nordica	SRR7174505	(Li et al. 2019)
	Spioniades abbreviata	SRR7174370	(Li et al. 2019)
	Staphylus hayhurstii	SRR7174537	(Li et al. 2019)
	Tiana niger	SRR7174566	(Li et al. 2019)
	Timochares trifasciata	SRR7174461	(Li et al. 2019)

Subfamily	Species	Accession number	Reference
	Timochreon satyrus	SRR7174493	(Li et al. 2019)
	Tosta tosta	SRR7174431	(Li et al. 2019)
	Trina geometrina	SRR7174491	(Li et al. 2019)
	Viola violella	SRR7174571	(Li et al. 2019)
	Xenophanes tryxus	SRR7174489	(Li et al. 2019)
	Zera sp.	SRR7174342	(Li et al. 2019)
	Zopyrion sandace	SRR7174530	(Li et al. 2019)
Papilionidae	*Papilio machaon*	NC_018047	Unpublished
	Papilio helenus	NC_025757	(Tang et al. 2014)
	Graphium timur	NC_024098	(Chen et al. 2016)
	Parnassius apollo	NC_024727	(Chen et al. 2014)

Table S2. Species information and GenBank accession numbers of *EF-1α* and *Wingless*

Species	Accession number		Reference	
	EF-1α	***Wingless***	***EF-1α***	***Wingless***
Astictopterus jama	OR030951	OR050643	This study	This study
Aethilla_lavochrea	EU364164.1	EU363971.1	(Warren et al. 2008)	(Warren et al. 2008)
Ampittia trimacula	MH310252.1	MH310318.1	(Huang et al. 2019)	(Huang et al. 2019)
Antigonus erosus	EU364160.1	EU363967.1	(Warren et al. 2008)	(Warren et al. 2008)
Atarnes sallei	EU364157.1	EU363964.1	(Warren et al. 2008)	(Warren et al. 2008)
Camptopleura auxo	EU364167.1	EU363974.1	(Warren et al. 2008)	(Warren et al. 2008)
Carcharodus alceae	EU364175.1	EU363982.1	(Warren et al. 2008)	(Warren et al. 2008)
Carterocephalus argyrostigma	Mitogenomes	Mitogenomes	This study	This study
Celaenorrhinus consanguineus	Mitogenomes	Mitogenomes	This study	This study
Celaenorrhinus maculosus	OR030953	OR050644	This study	This study
Coladenia agnioides	Mitogenomes	Mitogenomes	This study	This study
Charidia lucaria	KY014343.1	KY019961.1	(Sahoo et al. 2016)	(Sahoo et al. 2016)
Chiomara georgina	KY014344.1	KY019962.1	(Sahoo et al. 2016)	(Sahoo et al. 2016)
Cyclosemia anastomosis	EU364148.1	EU363955.1	(Warren et al. 2008)	(Warren et al. 2008)
Ebrietas anacreon	EU364169.1	EU363976.1	(Warren et al. 2008)	(Warren et al. 2008)
Eburuncus unifasciata	KY014354.1	SRR7174345	(Sahoo et al. 2016)	(Li et al. 2019)
Erynnis brizo brizo	SRR7174469	EU442874.1	(Li et al. 2019)	(Li et al. 2019)
Erynnis pelias	Mitogenomes	Mitogenomes	This study	This study
Gerosis phisara	OR030956	OR050645	This study	This study
Gorgythion begga	EU364152.1	EU363959.1	(Warren et al. 2008)	(Warren et al. 2008)
Hasora vitta	OR030958	OR050646	This study	This study
Heteropterus morpheus	OR030959	OR050647	This study	This study
Isoteinon lamprospilus	OR030960	OR050648	This study	This study
Lobocla bifasciata	OR030961	OR373070	This study	This study
Milanion marciana	EU364156.1	EU364156.1	(Warren et al. 2008)	(Warren et al. 2008)
Mooreana trichoneura	OR030962	OR050649	This study	This study
Netrocoryne repanda	EU364136.1	EU363943.1	(Warren et al. 2008)	(Warren et al. 2008)
Odontoptilum angulatum	KY014382.1	KY019994.1	(Sahoo et al. 2016)	(Sahoo et al. 2016)
Papilio helenus	AY457619.1	AY569121.1	This study	This study
Papilio machaon	EF485106.1	AY569124.1	This study	This study
Passova gellias	EU364107.1	EU363914.1	(Warren et al. 2008)	(Warren et al. 2008)
Pyrgus alveus speyeri	Mitogenomes	Mitogenomes	This study	This study
Pintara bowringi	Mitogenomes	Mitogenomes	This study	This study
Pyrgus maculatus	OR030964	OR373069	This study	This study
Pyrgus malvae	GU828922.1	GU829483.1	(Mutanen et al. 2010)	(Mutanen et al. 2010)
Pyrgus scriptura	EU364178.1	EU363985.1	(Warren et al. 2008)	(Warren et al. 2008)
Quadrus cerialis	EU364153.1	EU363960.1	(Warren et al. 2008)	(Warren et al. 2008)

Species	Accession number		Reference	
Sarangesa dasahara	OR030965	OR030948	This study	This study
Satarupa nymphalis	OR030966	OR030949	This study	This study
Sloperia tessellum dilutior	Mitogenomes	Mitogenomes	This study	This study
Sostrata nordica	EU364155.1	EU363962.1	This study	This study
Sovia lucasii lucasii	Mitogenomes	Mitogenomes	This study	This study
Timochares trifasciata	EU364165.1	EU363972.1	(Warren et al. 2008)	(Warren et al. 2008)
Xenophanes tryxus	KY014417.1	KY020023.1	(Sahoo et al. 2016)	(Sahoo et al. 2016)
Zopyrion sandace	EU364162.1	EU363969.1	(Warren et al. 2008)	(Warren et al. 2008)

Table S3. The geographic information used for this study

ID	Name	state
1	*Cecropterus lyciades*	B
2	*Eantis pallida*	AB
3	*Aethilla lavochrea*	AB
4	*Ampittia trimacula*	CE
5	*Anastrus sempiternus*	AB
6	*Anisochoria polysticta*	A
7	*Antigonus erosus*	AB
8	*Apostictopterus fuliginosus*	C
9	*Arteurotia tractipennis*	AB
10	*Astictopterus jama*	ACE
11	*Atarnes sallei*	AB
12	*Austinus heroica*	B
13	*Barca bicolor*	CE
14	*Bolla brennus*	AB
15	*Burnsius albescens*	AB
16	*Burnsius philetas*	B
17	*Camptopleura auxo*	AB
18	*Capila zennara*	E
19	*Carcharodus alceae*	CE
20	*Carrhenes canescens*	AB
21	*Carterocephalus argyrostigma*	C
22	*Carterocephalus silvicola*	BC
23	*Celaenorrhinus syllius*	A
24	*Celanorrhinus consanguineus*	A
25	*Celanorrhinus maculosus*	AC
26	*Charidia lucaria*	A
27	*Chiomara georgina*	AB
28	*Chirgus limbata*	AB
29	*Clito sp Burns02*	AB
30	*Coladenia agnoides*	E
31	*Cornuphallus problematica*	A
32	*Croniades pieria auraria*	A
33	*Cyclosemia anastomosis*	AB
34	*Doberes anticus*	ABC
35	*Eantis tamenenuned*	A
36	*Ebrietas anacreon*	AB
37	*Eburuncus unifasciata*	AB
38	*Ephyriades brunnea brunnea*	AB
39	*Eracon sarahburnsae*	A
40	*Eretis melania*	D
41	*Eyrnnis brizo brizo*	B
42	*Erynnis pelias*	C

ID	Name	state
43	*Euschemon rafflesia*	CF
44	*Gerosis phisara*	CE
45	*Gesta gesta*	AB
46	*Gindanes brontinus*	AB
47	*Gorgopas trochilus*	A
48	*Gorgythion begga*	AB
49	*Grais stigmaticus*	AB
50	*Graphium timur*	C
51	*Hasora badra*	CE
52	*Hasora vitta*	CE
53	*Helias cama*	AB
54	*Heliopetes ericetorum*	AB
55	*Heliopetes sublinea*	B
56	*Hesperopsis alpheus texana*	B
57	*Heteropterus morpheus*	C
58	*Isoteinon lamprospilus*	C
59	*Jera tricuspidata*	A
60	*Leptalina unicolor*	C
61	*Lerema accius*	AB
62	*Lobocla bifasciata*	CE
63	*Megathymus ursus violae*	B
64	*Metardaris cosinga cedra*	A
65	*Milanion marciana*	A
66	*Mimia cf. chiapaensis*	A
67	*Mooreana trichoneura*	CE
68	*Morvina fissimacula*	A
69	*Mylon lassia*	AB
70	*Myrinia cf binoculus*	A
71	*Netrocoryne repanda*	CDF
72	*Noctuana haematospila*	A
73	*Notocrypta curvifascia*	CE
74	*Ochlodes venata*	C
75	*Odontoptilum angulatum*	C
76	*Ouleus salvina*	AB
77	*Oxynetra aureopecta*	A
78	*Paches loxus*	AB
79	*Papilio helenus*	CE
80	*Papilio machaon*	ABE
81	*Paramimus stigma*	A
82	*Parnara guttatus*	CE
83	*Parnassius apollo*	C
84	*Passova gellias*	A
85	*Pellicia arina*	AB

ID	Name	state
86	*Pholisora mejicanus*	B
87	*Pintara bowringi*	C
88	*Plumbago plumbago*	A
89	*Polyctor polyctor*	AB
90	*Potamanaxas melicertes*	A
91	*Potanthus flavus*	CE
92	*Pseudocoladenia dan fabia*	E
93	*Pseudodrephalys atinas*	A
94	*Pyrgus alveus speyeri*	C
95	*Pyrgus maculatus*	C
96	*Pyrgus malvae*	AB
97	*Pyrgus scriptura*	ABE
98	*Pyrrhopyge hadassa*	A
99	*Pythonides amaryllis*	AB
100	*Quadrus cerialis*	AB
101	*Sarangesa dasahara*	CE
102	*Satarupa nymphalis*	C
103	*Sloperia tessellum dilutior*	C
104	*Sostrata nordica*	AB
105	*Sovia lucasii lucasii*	CE
106	*Spioniades abbreviata*	A
107	*Staphylus hayhurstii*	AB
108	*Tagiades menaka*	CE
109	*Tiana niger*	A
110	*Timochares trifasciata*	AB
111	*Timochreon satyrus*	A
112	*Tosta tosta*	A
113	*Trina geometrina*	A
114	*Viola violella*	A
115	*Xenophanes tryxus*	AB
116	*Zera sp.*	AB
117	*Zonia zonia panamensis*	A
118	*Zopyrion sandace*	AB

According to the distribution of the subfamily Pyrginae, we recognized the following six areas: A: Neotropical region B: Nearctic region C: Palearctic region D: Afrotropical region E: Indo-Malayan region F. Australasian region

Table S4 The best partitioning schemes and models for Maximum likelihood (ML) method based on PRTN data set selected by Model Finder.

Partition	Subset Partitions	Model
rrnL_gb_codon1_nad4L_mafft_gb_codon2	1-956\3, 9846-10114\3	TVM+F+I+R4
rrnL_gb_codon2_rrnS_gb_codon2_nad4L_mafft_gb_codon1	2-956\3, 958-1524\3, 9845-10114\3	GTR+F+R6
rrnL_gb_codon3	3-956\3	GTR+F+I+R5
rrnS_gb_codon1	957-1524\3	TPM3u+F+R5
rrnS_gb_codon3_trnH_gb_codon3_trnM_gb_codon1_trnY_gb_codon1_trnY_gb_codon2	959-1524\3, 1837-1857\3, 2098-2158\3, 2613-2671\3, 2614-2671\3	GTR+F+R4
trnA_gb_codon1_nad3_mafft_gb_codon1	1525-1569\3, 9500-9844\3	GTR+F+R4
trnA_gb_codon2_trnA_gb_codon3_trnD_gb_codon2_trnI_gb_codon2_trnR_gb_codon1_trnW_gb_codon1_trnY_gb_codon3	1526-1569\3, 1527-1569\3, 1629-1683\3, 1859-1907\3, 2338-2395\3, 2556-2612\3, 2615-2671\3	TVM+F+I+R2
trnC_gb_codon1_trnD_gb_codon1_trnV_gb_codon1	1570-1627\3, 1628-1683\3, 2520-2555\3	TVM+F+R3
trnC_gb_codon2_trnP_gb_codon1	1571-1627\3, 2216-2273\3	GTR+F+R3
trnC_gb_codon3	1572-1627\3	K3Pu+F+R3
trnD_gb_codon3_trnG_gb_codon1_trnG_gb_codon3_trnT_gb_codon2	1630-1683\3, 1785-1834\3, 1787-1834\3, 2479-2519\3	GTR+F+I+R3
trnE_gb_codon1_trnM_gb_codon2_trnR_gb_codon2_trnR_gb_codon3	1684-1727\3, 2099-2158\3, 2339-2395\3, 2340-2395\3	TVM+F+I+R2
trnE_gb_codon2_trnF_gb_codon1_trnQ_gb_codon2_trnS1_gb_codon2	1685-1727\3, 1728-1784\3, 2275-2337\3, 2397-2436\3	TIM+F+G4
trnE_gb_codon3_trnS1_gb_codon1	1686-1727\3, 2396-2436\3	GTR+F+G4

Partition	Subset Partitions	Model
trnF_gb_codon2_trnQ_gb_codon1	1729-1784\3, 2274-2337\3	TIM3+F+R3
trnF_gb_codon3_trnK_gb_codon3	1730-1784\3, 1910-1971\3	GTR+F+G4
trnG_gb_codon2_trnN_gb_codon1_trnN_gb_codon3_trnP_gb_codon2	1786-1834\3, 2159-2215\3, 2161-2215\3, 2217-2273\3	GTR+F+I+G4
trnH_gb_codon1_trnI_gb_codon3_trnL2_gb_codon3	1835-1857\3, 1860-1907\3, 2035-2097\3	TIM2+F+I+R3
trnH_gb_codon2_trnI_gb_codon1_trnM_gb_codon3_trnV_gb_codon2_123WG_mafft_gb_codon2	1836-1857\3, 1858-1907\3, 2100-2158\3, 2521-2555\3, 14316-14713\3	K3P+R2
trnK_gb_codon1	1908-1971\3	TIM3+F+I+R3
trnK_gb_codon2_cox3_mafft_gb_codon1	1909-1971\3, 5666-6439\3	GTR+F+I+G4
trnL1_gb_codon1_trnL1_gb_codon3	1972-2032\3, 1974-2032\3	TN+F+G4
trnL1_gb_codon2_trnQ_gb_codon3	1973-2032\3, 2276-2337\3	TN+F+I+G4
trnL2_gb_codon1_trnP_gb_codon3_trnT_gb_codon3_trnV_gb_codon3_trnW_gb_codon2_trnW_gb_codon3	2033-2097\3, 2218-2273\3, 2480-2519\3, 2522-2555\3, 2557-2612\3, 2558-2612\3	TIM+F+R3
trnL2_gb_codon2_trnT_gb_codon1	2034-2097\3, 2478-2519\3	GTR+F+R3
trnN_gb_codon2_cox1_mafft_gb_codon2_cox2_mafft_gb_codon2	2160-2215\3, 3492-5020\3, 5022-5665\3	TVM+F+R3
trnS1_gb_codon3_trnS2_gb_codon1_trnS2_gb_codon2_trnS2_gb_codon3	2398-2436\3, 2437-2477\3, 2438-2477\3, 2439-2477\3	GTR+F+R3
atp6_mafft_gb_codon1	2672-3340\3	GTR+F+R5
atp6_mafft_gb_codon2	2673-3340\3	GTR+F+I+R3
atp6_mafft_gb_codon3	2674-3340\3	TIM+F+I+R5
atp8_mafft_gb_codon1	3341-3490\3	TN+F+I+G4

Partition	Subset Partitions	Model
atp8_mafft_gb_codon2	3342-3490\3	GTR+F+I+G4
atp8_mafft_gb_codon3	3343-3490\3	K3Pu+F+R4
cox1_mafft_gb_codon1	3491-5020\3	GTR+F+I+R5
cox1_mafft_gb_codon3_cox2_mafft_gb_codon3	3493-5020\3, 5023-5665\3	TIM+F+R7
cox2_mafft_gb_codon1	5021-5665\3	GTR+F+I+R4
cox3_mafft_gb_codon2	5667-6439\3	TIM+F+R4
cox3_mafft_gb_codon3	5668-6439\3	TPM2u+F+I+R8
cytb_mafft_gb_codon1	6440-7576\3	GTR+F+I+R4
cytb_mafft_gb_codon2	6441-7576\3	GTR+F+I+R3
cytb_mafft_gb_codon3	6442-7576\3	TPM2u+F+I+R7
nad1_mafft_gb_codon1_nad5_mafft_gb_codon1	7577-8503\3, 11447-13150\3	GTR+F+I+R6
nad1_mafft_gb_codon2	7578-8503\3	GTR+F+R4
nad1_mafft_gb_codon3	7579-8503\3	TVM+F+R6
nad2_mafft_gb_codon1	8504-9499\3	TIM3+F+R5
nad2_mafft_gb_codon2	8505-9499\3	GTR+F+I+R4
nad2_mafft_gb_codon3	8506-9499\3	TVM+F+R7
nad3_mafft_gb_codon2	9501-9844\3	TVM+F+I+R3
nad3_mafft_gb_codon3	9502-9844\3	K3Pu+F+R6
nad4L_mafft_gb_codon3	9847-10114\3	K3Pu+F+R4
nad4_mafft_gb_codon1	10115-11446\3	GTR+F+R5
nad4_mafft_gb_codon2	10116-11446\3	GTR+F+I+R4
nad4_mafft_gb_codon3	10117-11446\3	GTR+F+I+R5
nad5_mafft_gb_codon2	11448-13150\3	GTR+F+R5
nad5_mafft_gb_codon3	11449-13150\3	K3Pu+F+I+R7
nad6_mafft_gb_codon1	13151-13678\3	TIM2+F+I+R5
nad6_mafft_gb_codon2	13152-13678\3	TPM3u+F+R5
nad6_mafft_gb_codon3	13153-13678\3	TIM2+F+R6
EF_mafft_gb_codon1	13679-14314\3	TN+F+I+R3
EF_mafft_gb_codon2	13680-14314\3	TVM+F+I+R2
EF_mafft_gb_codon3	13681-14314\3	SYM+I+R4

Partition	Subset Partitions	Model
WG_mafft_gb_codon1	14315-14713\3	GTR+F+R3
WG_mafft_gb_codon3	14317-14713\3	SYM+I+R4

Table S5 The best partitioning schemes and models for Bayesian inference (BI) method based on PRTN dataset selected by Model Finder.

Partition	Subset Partitions	Model
rrnL_gb_rrnS_gb_trnH_gb	1-956, 957-1524, 1835-1857	GTR{0.319818,6.40585,2.98322,0.111686,3.21975} + F{0.408765,0.0633533,0.122874,0.405008} + I{0.418973} + G4{0.483104} {6.71969}
trnA_gb_trnE_gb_trnG_gb_trnN_gb_trnR_gb_trnS1_gb_trnS2_gb_trnT_gb	1525-1569, 1684-1727, 1785-1834, 2159-2215, 2338-2395, 2396-2436, 2437-2477, 2478-2519	GTR{1.475,4.86248,10.111,0.0001,20.0893} + F{0.401766,0.0916186,0.0954976,0.411118} + I{0.326879} + G4{0.408568} {7.81975}
trnC_gb_trnD_gb_nad4L_mafft_gb_nad4_mafft_gb	1570-1627, 1628-1683, 9845-10114, 10115-11446	GTR{0.602338,12.9579,3.03021,3.66709,5.65728} + F{0.334786,0.0652624,0.122405,0.477546} + I{0.292439} + G4{0.586894} {17.1952}
trnF_gb	1728-1784	HKY{12.783} + F{0.428945,0.0271931,0.144876,0.398986} + G4{0.238451} {32.0664}
trnI_gb_trnK_gb_trnL2_gb_trnM_gb_trnP_gb_trnV_gb_trnW_gb_trnY_gb	1858-1907, 1908-1971, 2033-2097, 2098-2158, 2216-2273, 2520-2555, 2556-2612, 2613-2671	GTR{2.79382,26.9227,6.90276,0.0001,22.3149} + F{0.385316,0.103516,0.126564,0.384604} + I{0.456198} + G4{0.361089} {4.01298}
trnL1_gb_trnQ_gb	1972-2032, 2274-2337	GTR{0.333832,13.6474,0.871902,0.0001,2.3525} + F{0.406475,0.0491927,0.119436,0.424897} + I{0.206604} + G4{0.518642} {14.3594}
atp6_mafft_gb_cox2_mafft_gb_cytb_mafft_gb_nad3_mafft_gb	2672-3340, 5021-5665, 6440-7576, 9500-9844	GTR{6.12855,13.4844,14.2612,4.47226,100} + F{0.341705,0.133887,0.0927576,0.43165} + I{0.373809} + G4{0.488624} {23.6867}
atp8_mafft_gb_nad6_mafft_gb	3341-3490, 13151-13678	GTR{6.37832,5.66224,3.80521,3.84489,38.6278} + F{0.384985,0.0822396,0.046654,0.486121} + I{0.177629} + G4{0.568323} {31.78}

Partition	Subset Partitions	Model
cox1_mafft_gb_cox3_mafft_gb	3491-5020, 5666-6439	GTR{4.15465,12.5657,21.6427,4.2754,100} + F{0.317122,0.144233,0.128603,0.410042} + I{0.457522} + G4{0.567659} {16.105}
nad1_mafft_gb_nad5_mafft_gb	7577-8503, 11447-13150	GTR{0.161845,10.5805,2.06669,3.92249,2.22424} + F{0.326717,0.0662865,0.122187,0.484809} + I{0.308045} + G4{0.604085} {18.9446}
nad2_mafft_gb	8504-9499	GTR{2.18314,5.73696,2.96,4.04741,19.4225} + F{0.35122,0.0908983,0.0617293,0.496152} + I{0.294081} + G4{0.581421} {19.139}
EF_mafft_gb	13679-14314	SYM{1.69824,7.49573,3.22143,1.01062,11.9005} + FQ + I{0.511227} + G4{0.736986} {16.7434}
WG_mafft_gb	14315-14713	GTR{0.914711,3.27458,1.03884,0.593361,5.73294} + F{0.212204,0.289468,0.31456,0.183768} + I{0.379246} + G4{0.751277} {9.65798}

Table S6 The AU test result based on IQTREE

Tree	logL	deltaL	bp-RELL	p-KH	p-SH	p-WKH	p-WSH	c-ELW	p-AU
ML	-425309	0	0.578+	0.587+	1+	0.587+	0.587+	0.578+	0.587+
BI	-425313	3.9925	0.422+	0.413+	0.413+	0.413+	0.413+	0.422+	0.413+

Figure S1 Phylogenetic tree inferred from the PRTN dataset using maximum likelihood method

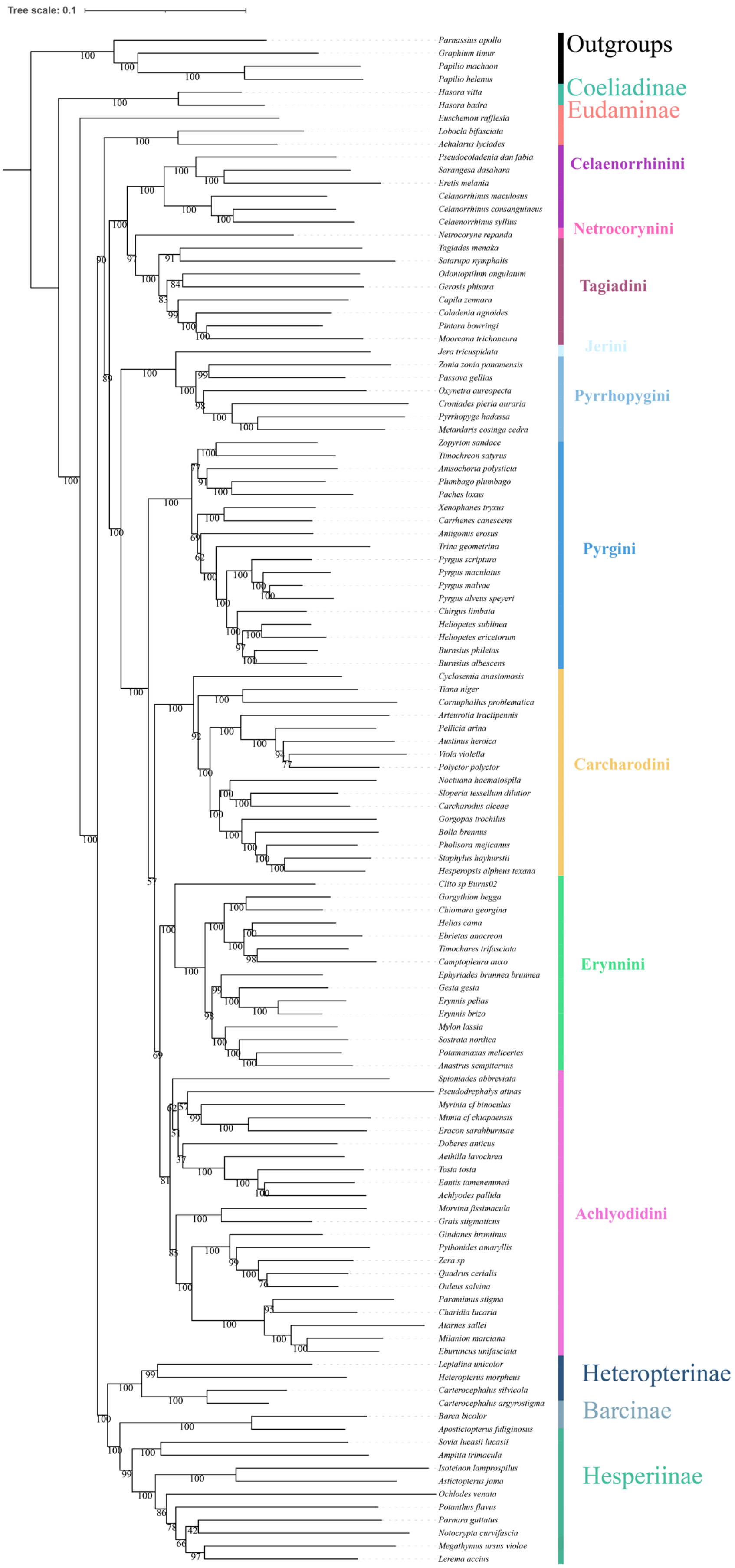

Figure S2 Species tree inferred from the PRTN dataset using ASTRAL-III

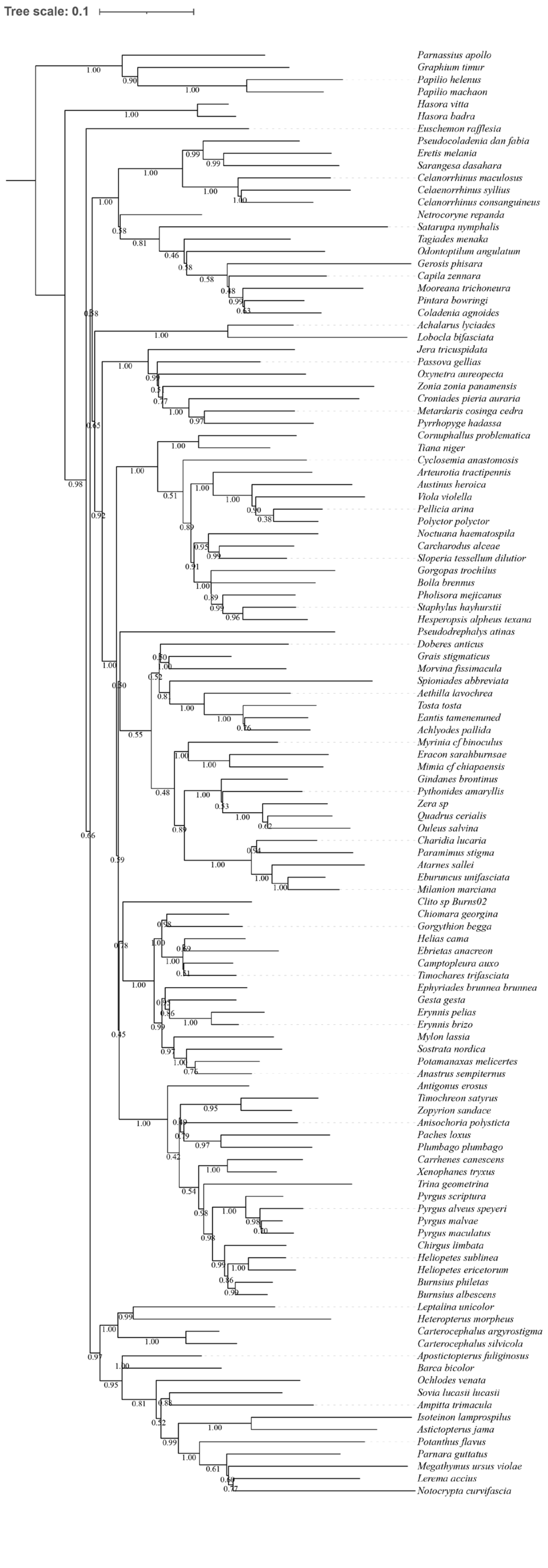

Figure S3 95% HPD ranges for node ages of the time tree based on PRTN dataset using Bayesian analysis

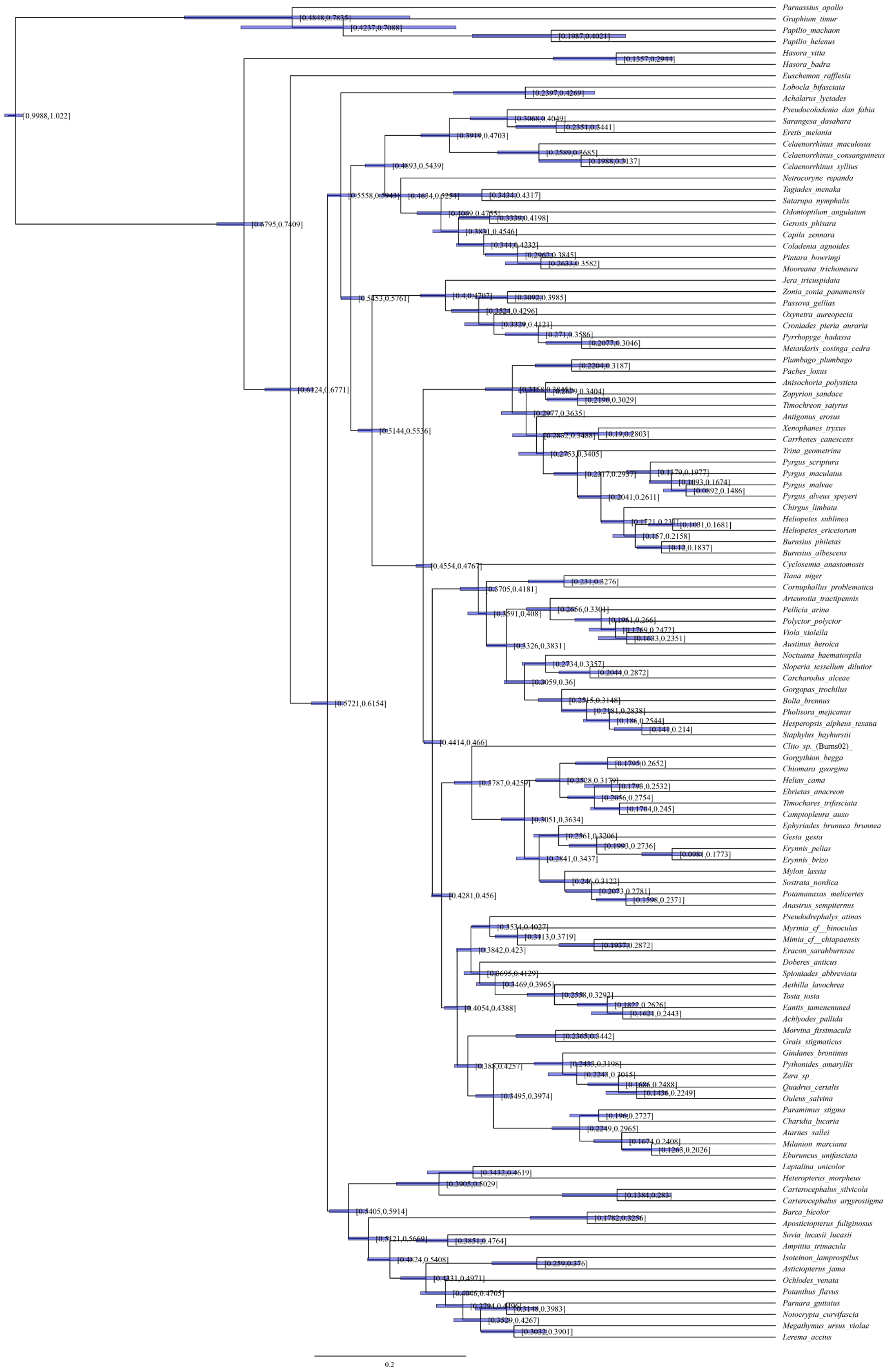

Figure S4 The node ages of the time tree based on PRTN dataset using Bayesian analysis

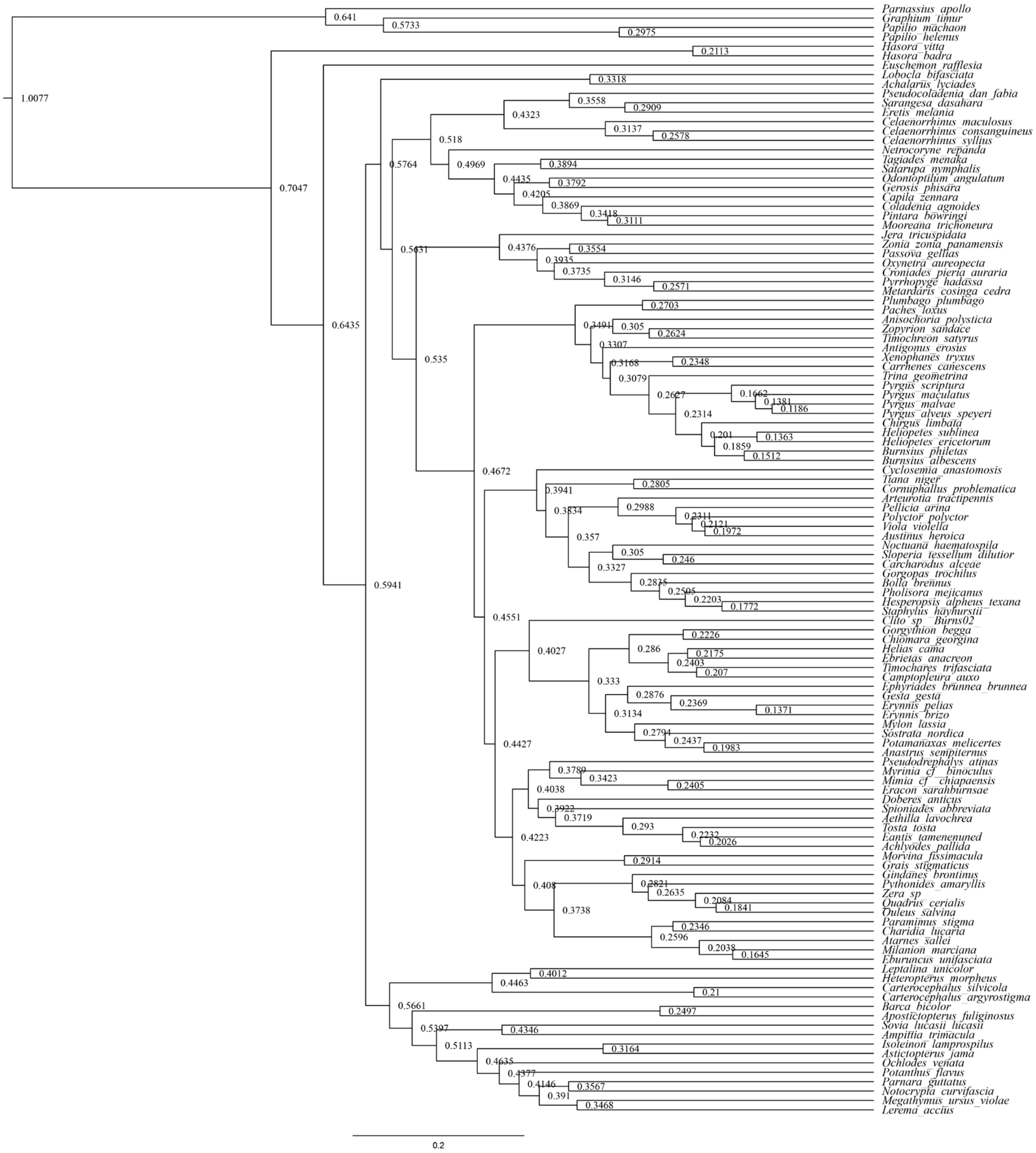

Supplement: Supplementary file 1 — Data S1. [file ECE3-15-e71757-s001.pdf]
